# Prevalence of, and factors associated with, sarcopenia in Iran: a systematic review and meta-analysis

**DOI:** 10.3389/fnut.2024.1457768

**Published:** 2025-01-07

**Authors:** Mobin Marzban Abbas Abadi, Danial Hosseinzade, Majid Khalilizad

**Affiliations:** ^1^Department of Orthopedic and Trauma Surgery, Babol University of Medical Sciences, Babol, Iran; ^2^Mobility Impairment Research Center, Health Research Institute, Babol University of Medical Sciences, Babol, Iran; ^3^Sport and Reconstruction Surgery, Clinical Research Development Unit of Shahid Beheshti Hospital, Babol University of Medical Sciences, Babol, Iran

**Keywords:** sarcopenia, prevalence, risk factor, systematic review, Iran

## Abstract

**Background:**

Several studies have assessed the epidemiology of sarcopenia in Iran with conflicting results, but comprehensive information has remained limited. Therefore, we aimed to perform a systematic review and meta-analysis of the prevalence of sarcopenia and its associated factors among Iranian population.

**Methods:**

We searched in Embase, PubMed, Scopus, and Google Scholar, as well as Persian databases including the Scientific Information Database and Magiran, from inception to 31 May 2024. We included cross-sectional observational studies reporting the prevalence rate of, and/or factors associated with, sarcopenia in Iranian population. The pooled prevalence of sarcopenia was estimated using a random-effects model.

**Results:**

Totally, 14 eligible studies were included. The overall pooled prevalence of sarcopenia in Iran was 22.1% (95% confidence interval [CI]: 18.7–25.6). This rate in older adult population (≥60 years) was 23.5% (95% CI: 17.9–29.0). Sarcopenia was more prevalent in men (23.2% [95% CI: 21.3–25.1]) than in women (15.5% [95% CI: 9.2–21.9]). The prevalence of severe sarcopenia was 24.5% (95% CI: 16.9–32.0). Factors associated with an increased risk of sarcopenia included older age, male gender, higher body fat, lower socioeconomic status, lower education, insufficient physical activity, diabetes, smoking, and malnutrition; conversely, dietary patterns rich in anti-inflammatory nutrients, Mediterranean diet adherence, and higher mineral and vitamin intake were associated with a lower likelihood of sarcopenia.

**Conclusion:**

The prevalence of sarcopenia is notably high in Iran, particularly among men and older ages. These findings emphasize the need for targeted interventions in nutrition and lifestyle to reduce sarcopenia risk and improve quality of life among Iranians.

## Introduction

Sarcopenia, characterized by the progressive loss of skeletal muscle mass and strength, has emerged as a significant public health concern, particularly among older adults. This condition not only leads to physical disabilities and reduced quality of life but also raises mortality and charges a substantial financial strain on healthcare systems (1, 2). Despite growing global awareness, the prevalence and factors associated with sarcopenia exhibit considerable variation across different populations and regions, necessitating localized studies to inform targeted interventions (3, 4).

Iran, a country with a rapidly aging population, faces unique demographic and epidemiological transitions that may influence the prevalence and determinants of sarcopenia (5, 6). It is crucial to comprehend these dynamics in order to develop customized and successful public health strategies tailored to the Iranian context. Until now, several studies have been conducted to explore the epidemiology of sarcopenia among the Iranian population with various results, ranging from 16 to 32% reported for the prevalence (7, 8); however, comprehensive data on this subject has remained sparse and fragmented.

Therefore, the present systematic review and meta-analysis aimed to address this knowledge gap by synthesizing available evidence on the prevalence of sarcopenia and identifying its associated factors among the Iranian population. By integrating data from various studies, we seek to provide robust estimates of sarcopenia prevalence and elucidate key factors that may contribute to the risk of developing sarcopenia in Iran. Our findings will inform policymakers, clinicians, and researchers on effective interventions and prevention strategies for sarcopenia in the Iranian population. The study also will underscore regional variations, emphasizing the importance of culturally relevant approaches to address this health issue globally.

## Methods

### Study outcome

Our primary outcome was the prevalence of sarcopenia in the Iranian population. The secondary outcome of our study was factors potentially associated with sarcopenia in Iran.

### Search strategy

We followed the PRISMA (Preferred reporting items for systematic review and meta-analysis) guideline to present this systematic review and meta-analysis (9). A literature search was done in PubMed, Embase, Scopus, and Google Scholar, considering publications from the inception to 31 May 2024 without language limitations. We utilized the following keywords for database search, applying to Title/Abstract: (“sarcopenia” OR “sarcopenic”) AND (“Iran” OR “Iranian” OR “Iranians”). The search strategy has been fully mentioned in the Supplement. The Iranian databases, including the Scientific Information Database and Magiran, were also searched using the Persian equivalents of the keywords. Furthermore, additional sources were sought by examining the reference lists of related reviews and the enrolled papers.

### Eligibility criteria

We included cross-sectional observational studies that reported the prevalence of sarcopenia in the Iranians (*n* ≥ 50 participants) and examined the factors related to it. We took into account various diagnostic criteria for sarcopenia as long as supported by reliable evidence. Our study included individuals of all ages and both sexes. The following criteria were used to exclude studies from this systematic review: (1) reviews, case reports, editorials, and letters to the editor; (2) duplicate publications; (3) surveys involving only subjects with special conditions (e.g., dialysis, obesity, etc.); (4) research lacking extractable information on our outcome; (5) unavailable full-texts.

### Study selection and data extraction

The MMAA and DH initially reviewed article titles and abstracts and then obtained potential full-texts to evaluate their suitability for inclusion; consensus was held to resolve disagreements. Finally, MMAA and DH collected the following data from the included studies: first author’s name, publication date, location of study, study period, characteristics of the participants (including sample size, sex, and age), prevalence rate of sarcopenia, diagnostic criteria for sarcopenia, factors associated with sarcopenia risk (demographic, socioeconomic, lifestyle, health status, nutritional, etc.). We extracted information on the risk factors pre-specified based on prior knowledge in the literature and derived from confounder-adjusted analyses in the original studies enrolled. We reached out to authors if full texts or necessary data were unavailable. Google Translate was used for non-English and non-Persian reports. We considered separate data for sarcopenia classification as different reports for analysis. Lastly, in cases where duplicate publications on the same population were identified, we selected the one that provided more detailed information or had a larger sample size for inclusion.

### Risk of bias assessment

For our primary outcome, MMAA and DH used a checklist developed by Hoy et al. (10) to assess the quality of studies; consensus was held to resolve any discrepancies. The checklist encompasses nine criteria with two response options (Yes/No). Studies are categorized as “high quality” (scores 0–3), “moderate quality” (scores 4–6), or “low quality” (scores 7–9) based on their scores.

### Statistical analysis

We estimated the prevalence of sarcopenia by combining data from the individual articles using a random-effects model to provide more conservative estimates irrespective of the heterogeneity index. The pooled prevalence rates were presented with percentages and 95% confidence intervals (CIs). Subgroup analyses were conducted based on diagnostic criteria, sex, study period, study quality, study location, and sample size, with a *p*-value <0.10 considered statistically significant for differences between subgroups (‘*p* for interaction’) (11). We categorized the study dates as <2015 and ≥2015 based on the distribution of reports in each period. As sensitivity analyses, we performed meta-analyses on studies involving the older adult population (aged ≥60 years) and those carried out on severe sarcopenia. We also conducted a leave-one-out sensitivity analysis to evaluate the robustness of our meta-analytic estimates. We used the *I*^2^ index (ranging from 0% [lowest] to 100% [highest]) to measure the heterogeneity among the studies and the chi-squared test (*p*-value <0.10) to identify significant heterogeneity. Meta-regression was also carried out to assess whether the use of different diagnostic criteria for sarcopenia affects our study outcome, with a *p*-value <0.05 revealing a statistical significance. Publication bias was examined with a funnel plot and the Egger’s test, with a *p*-value <0.05 indicating a significant result. All statistical analyses were conducted using STATA (StataCorp, College Station, TX, United States).

## Results

### Study selection and characteristics

**Figure 1:**
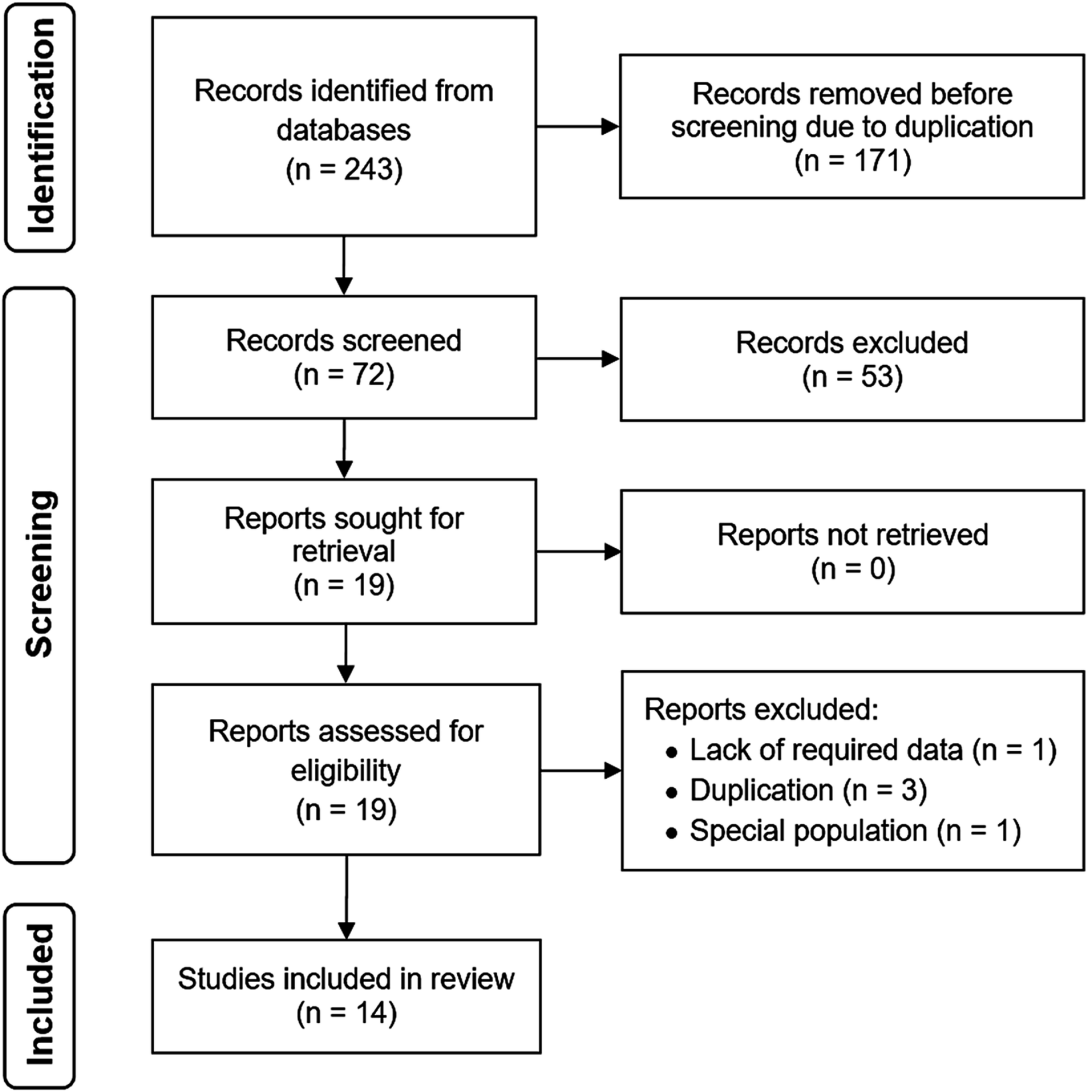
PRISMA flow diagram.

Of the 243 citations initially yielded, 72 were evaluated after removing duplicates. After the title and abstract review, 53 articles were excluded. Further assessment of full-texts resulted in 5 more articles being deemed ineligible. Ultimately, 14 research articles met the inclusion criteria (7, 8, 12–23). Figure 1 denotes the PRISMA chart, visually representing the search strategy results. The enrolled studies were conducted in Bushehr, Ravansar, Shiraz, and Tehran, published between 2016 and 2024.

### Pooled prevalence of sarcopenia

The characteristics of 8 papers reporting the prevalence of sarcopenia (comprising 8,856 participants; 53.5% women) from 4 regions (Bushehr [one study], Ravansar [one study], Shiraz [one study], and Tehran [five studies]) are summarized in Table 1. There were six high-quality and two moderate-quality studies; the risk of bias appraisal has been represented in Supplementary Table S1 in detail. Criteria used to define sarcopenia included EWGSOP (European Working Group on Sarcopenia in Older People), AWGS (Asian Working Group for Sarcopenia), FNIH (Foundation for the National Institutes of Health), and SARC-F (Strength, Assistance walking, Rising from a chair, Climbing stairs, Falls).

**Table 1:** Baseline characteristics of cross-sectional studies reporting prevalence of sarcopenia in Iran.

Study	Location	Period	Participant characteristics	Diagnostic criteria	Quality (score)
Dorosty (2016) (7)	Tehran	2014–2015	Total (*n*): 644 Mean age (years): 70.8 Men/women (*n*): 310/334	EWGSOP, AWGS, FNIH	High (3)
Hashemi (2016) (14)	Tehran	2011	Total (*n*): 300 Mean age (years): 66.8 Men/women (*n*): 150/150	EWGSOP	High (3)
Maghbooli (2017) (17)	Tehran	NA	Total (*n*): 409 Mean age (years): 56.5 Men/women (*n*): 0/409	SARC-F	Moderate (5)
Maghbooli (2022) (18)	Tehran	2016	Total (*n*): 305 Mean age (years): 57.9 Men/women (*n*): 0/305	EWGSOP	Moderate (4)
Mohseni (2017) (20)	Tehran	2015	Total (*n*): 250 Mean age (years): 57.5 Men/women (*n*): 0/250	EWGSOP	High (3)
Nasimi (2019) (21)	Shiraz	2017–2018	Total (*n*): 501 Mean age (years): 70.3 Men/women (*n*): 254/247	AWGS	High (3)
Pasdar (2022) (22)	Ravansar	2014	Total (*n*): 4,021 Mean age (years): 47.9 Men/women (*n*): 2,250/1,781	EWGSOP	High (1)
Shafiee (2020) (8)	Bushehr	2013–2014	Total (*n*): 2,426 Mean age (years): 69.3 Men/women (*n*): 1,166/1,260	EWGSOP 1 and 2	High (3)

EWGSOP, European Working Group on Sarcopenia in Older People; AWGS, Asian Working Group for Sarcopenia; FNIH, Foundation for the National Institutes of Health; NA, not applicable; SARC-F, Strength, Assistance walking, Rising from a chair, Climbing stairs, Falls.

**Figure 2:**
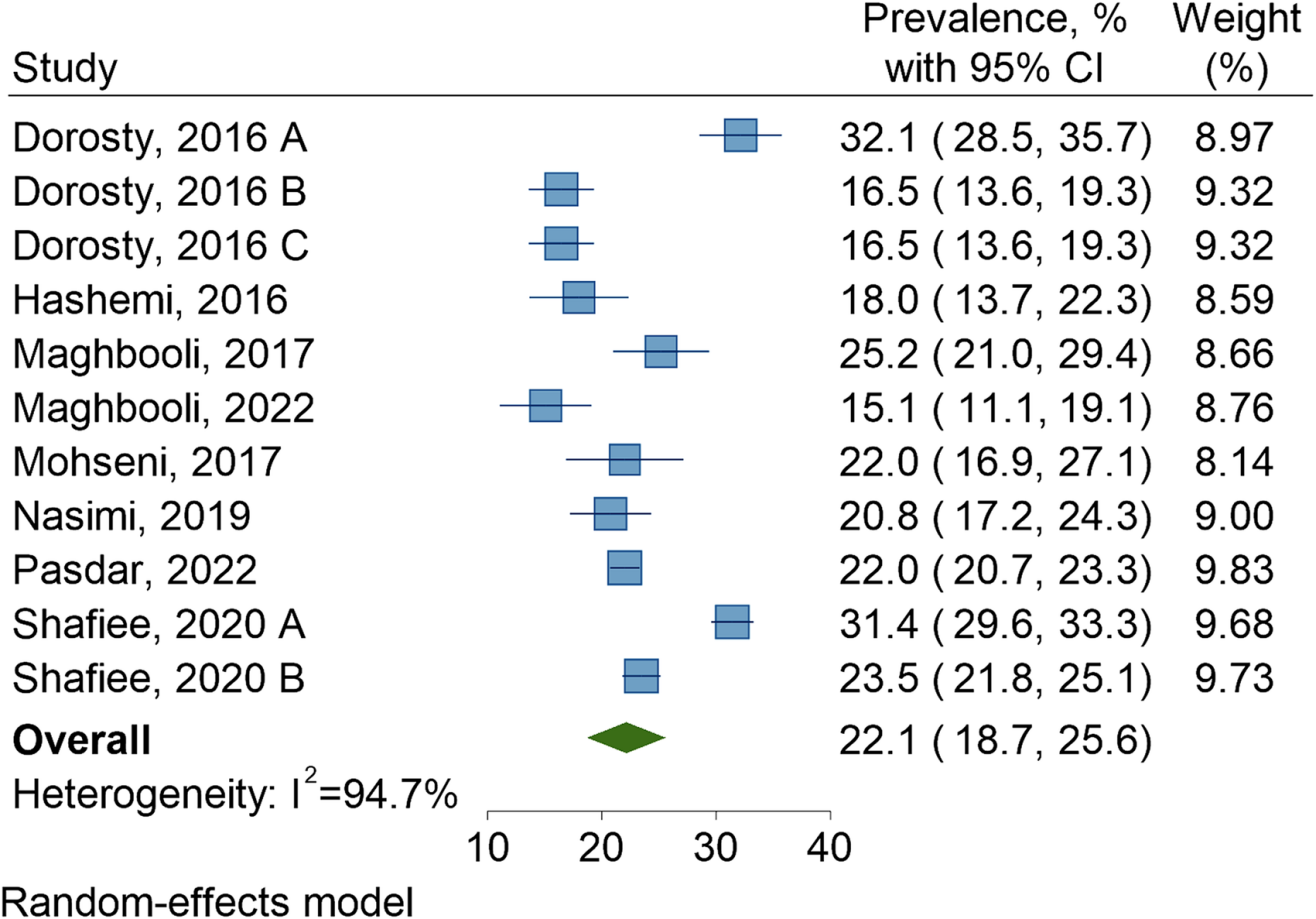
Pooled prevalence of sarcopenia in Iran.

The overall pooled prevalence of sarcopenia in Iran was 22.1% (95% CI: 18.7–25.6; *I*^2^ = 94.7%, *p* < 0.001) (Figure 2). The Funnel plot appeared asymmetrical (Supplementary Figure S1), but the Egger’s test showed no small-study effects (*p* = 0.515). In our sensitivity analysis employing the leave-one-out forest plot, we observed that the overall pooled effect estimate remained largely unchanged by any individual study (Supplementary Figure S2).

Table 2 represents the prevalence rate of sarcopenia according to different subgroups. This estimated rate was 18.5% (95% CI: 14.3–22.7) per AWGS, 23.6% (95% CI: 18.9–28.3) per EWGSOP, 16.5% (95% CI: 13.6–19.3) per FNIH, and 25.2% (21.0–29.4) per SARC-F (*p* for interaction = 0.002). Also, there was a significant difference in the prevalence of sarcopenia between men (23.2% [95% CI: 21.3–25.1]) and women (15.5% [95% CI: 9.2–21.9]) (*p* for interaction = 0.023). On the other hand, no significant differences were observed in the sarcopenia prevalence based on study period (<2015, 22.9% [95% CI: 18.0–27.8]; ≥2015, 20.7% [95% CI: 16.5–24.9]; *p* for interaction = 0.504), study quality (high, 22.6% [95% CI: 18.8–26.4]; moderate, 20.1% [95% CI: 10.2–30.0]; *p* for interaction = 0.649), study location (Bushehr, 27.4% [95% CI: 19.6–35.2]; Ravansar, 22.0% [95% CI: 20.7–23.3]; Shiraz, 20.8% [95% CI: 17.2–24.3]; Tehran, 20.7% [95% CI: 16.1–25.3]; *p* for interaction = 0.457), and sample size (*n* < 500, 20.0% [95% CI: 15.5–24.5]; 500 ≤ *n* ≤ 1,000, 21.4% [95% CI: 14.2–28.6]; *n* > 1,000, 25.6% [95% CI: 19.9–31.3]; *p* for interaction = 0.313).

**Table 2:** Pooled prevalence of sarcopenia according to different subgroups.

Subgroups	Studies (*n*)	Participants (*n*)	Prevalence, % (95% CI)	*I* ^2^	*P*-value for *χ*^2^	*P*-value for interaction
Overall	8	8,856	22.1 (18.7–25.6)	94.7%	<0.001	NA
Diagnostic criteria						
AWGS	2	1,145	18.5 (14.3–22.7)	70.7%	0.065	0.002
EWGSOP	6	7,946	23.6 (18.9–28.3)	96.3%	<0.001	
FNIH	1	644	16.5 (13.6–19.3)	NA	NA	
SARC-F	1	409	25.2 (21.0–29.4)	NA	NA	
Sex						
Men	4	1,880	23.2 (21.3–25.1)	0.0%	0.298	0.023
Women	4	1,991	15.5 (9.2–21.9)	92.1%	<0.001	
Study period						
<2015	4	7,391	22.9 (18.0–27.8)	97.1%	<0.001	0.504
≥2015	4	1,465	20.7 (16.5–24.9)	75.1%	0.007	
Study quality						
High	6	8,142	22.6 (18.8–26.4)	95.5%	<0.001	0.649
Moderate	2	714	20.1 (10.2–30.0)	91.4%	0.001	
Study location						
Bushehr	1	2,426	27.4 (19.6–35.2)	NA	NA	0.457
Ravansar	1	4,021	22.0 (20.7–23.3)	NA	NA	
Shiraz	1	501	20.8 (17.2–24.3)	NA	NA	
Tehran	5	1,908	20.7 (16.1–25.3)	90.8%	<0.001	
Sample size, *n*						
<500	4	1,264	20.0 (15.5–24.5)	75.7%	0.005	0.313
500–1,000	2	1,145	21.4 (14.2–28.6)	95.1%	<0.001	
>1,000	2	6,447	25.6 (19.9–31.3)	97.4%	<0.001	

CI, confidence interval; NA, not applicable; AWGS, Asian Working Group for Sarcopenia; EWGSOP, European Working Group on Sarcopenia in Older People; FNIH, Foundation for the National Institutes of Health; SARC-F, Strength, Assistance walking, Rising from a chair, Climbing stairs, Falls.

The sensitivity analysis demonstrated that the pooled prevalence of severe sarcopenia was 24.5% (95% CI: 16.9–32.0; *I*^2^ = 97.0%, *p* < 0.001) in Iran based on two studies (comprising 2,726 subjects), with no significant difference with the overall sarcopenia prevalence (*p* for interaction = 0.572). Additionally, the prevalence of sarcopenia in the older adult population was 23.5% (95% CI: 17.9–29.0; *I*^2^ = 96.6%, *p* < 0.001) according to three studies (encompassing 3,571 participants).

Meta-regression analysis demonstrated that different diagnostic criteria for sarcopenia did not significantly influence the outcome of interest (*β* = −0.975, *p* = 0.601).

### Factors associated with risk of sarcopenia

Table 3 summarizes the results of studies reporting factors associated with the risk of sarcopenia in Iran. There are various factors suggested by research papers to be linked to sarcopenia risk as follows:

- Demographic factors: older age, male gender, and higher body fat were associated with an increased risk of sarcopenia.
- Socioeconomic and lifestyle factors: poorer socioeconomic circumstances, lower educational levels, and insufficient physical activity were linked to higher odds of sarcopenia.
- Health status: diabetes, smoking, malnutrition, and low serum albumin levels were directly related to the risk of sarcopenia.
- Nutritional factors: adherence to an anti-inflammatory nutrient pattern, a Mediterranean diet, a healthier beverage pattern, and increased mineral and vitamin intake were inversely associated with the likelihood of sarcopenia.

**Table 3:** Studies reporting factors associated with risk of sarcopenia in Iran.

Study	Location	Period	Participants	Findings
Bagheri (2021) (12)	Tehran	2011	Total (*n*): 300 Mean age (years): 66.8 Men/women (*n*): 150/150	Adherence to the anti-inflammatory nutrient pattern characterized by high consumption of polyunsaturated fat, monounsaturated fat, copper, vitamin E, omega-3, magnesium, iron, pyridoxine (vitamin B6), sodium, and caffeine may reduce the odds of sarcopenia.
Bagheri, 2021 (13)				Consuming a diet with a higher Food-based Inflammatory Potential score, indicating a more pro-inflammatory diet, is positively associated with sarcopenia.
Hashemi (2016) (14)				Older age, male gender, and smoking are independently connected with sarcopenia.
Heshmat (2018) (15)	Bushehr	2013–2014	Total (*n*): 2,426 Mean age (years): 69.3 Men/women (*n*): 1,166/1,260	Sarcopenia is more prevalent among individuals with lower socioeconomic status.
Larijani, 2018 (16)				Sarcopenia is strongly inversely associated with the level of minerals, such as calcium, iron, magnesium, phosphorus, potassium, zinc, and vitamins A, E, C, biotin, B2, B3, and B6. Also, subjects with higher tertiles of daily protein intake, carbohydrates, and total calories exhibited a significantly decreased risk of sarcopenia.
Shafiee (2018) (23)				Older adult individuals with diabetes have a significantly higher risk of sarcopenia than those without.
Shafiee (2020) (8)				Low educational level, older age, high body fat, and lack of physical activity are linked to a greater sarcopenia risk.
Dorosty (2016) (7)	Tehran	2014–2015	Total (*n*): 644 Mean age (years): 70.8 Men/women (*n*): 310/334	The chance of sarcopenia is inversely associated with socioeconomic status.
Mahmoodi (2024) (19)	Shiraz	2017–2018	Total (*n*): 160 Mean age (years): 69.0 Men/women (*n*): 88/72	There is a negative association between the healthy beverage index and the odds of sarcopenia.
Nasimi (2019) (21)			Total (*n*): 501 Mean age (years): 70.3 Men/women (*n*): 254/247	The risk of sarcopenia increases with older age, male gender, decreased Mini-Nutritional Assessment score, lower serum albumin level, and higher fat mass.
Mohseni (2017) (20)	Tehran	2015	Total (*n*): 250 Mean age (years): 57.5 Men/women (*n*): 0/250	The Mediterranean diet pattern, including high consumption of olive oil, low-fat dairy, vegetables, fish, nuts, and vegetable oil, plays a beneficial role in preventing sarcopenia.

## Discussion

Sarcopenia can pose significant health risks and economic burdens, especially in aging populations such as that of Iran. While studies on sarcopenia epidemiology among Iranians showed varied results, comprehensive data remained limited, highlighting the need for targeted public health strategies informed by localized research. In the present study, we aimed to answer this query by performing a systematic review and meta-analysis on the prevalence of sarcopenia and its associated factors. After scrutinizing references initially identified from the scientific database search, a total of 14 eligible articles were ultimately retrieved.

Based on our analysis, the prevalence rate of sarcopenia in Iran was approximately 22%, which was considerably higher than that reported in a previous meta-analysis by Shafiee et al. (24) at the global scale (10%). On the other hand, our estimate was slightly lower than that Veronese et al. (25) estimated in Africa (26%). There were also variations in the sarcopenia prevalence we estimated according to different classifications. In this regard, a recent meta-analysis by Petermann-Rocha et al. (26) reported that the global prevalence rates of sarcopenia per EWGSOP, AWGS, and FNIH were 22, 15, and 11%, respectively; the authors also stated that the highest and lowest sarcopenia prevalence pertained to Oceania (40%) and Europe (1%) using the EWGSOP, respectively. Lastly, the prevalence of severe sarcopenia in their study ranged from 2 to 9%, which was lower than our findings (~12%) (26). The variation in prevalence according to different classifications highlights the importance of using standardized criteria in clinical practice to improve diagnosis and treatment.

Analysis of our data on the older adult population indicated a prevalence rate of roughly 24%, which is slightly higher than that reported by Whaikid and Piaseu (27) in Thai older people (~21%). Moreover, Petermann-Rocha et al. (26) stated that the sarcopenia prevalence ranged from 10 to 27% in the older adult worldwide. We also found that older age was associated with an increased risk of sarcopenia in Iran. This could be explained by age-related declines in muscle mass, strength, and function, compounded by factors like reduced physical activity and hormonal changes. These results underscore the clinical importance of monitoring and addressing sarcopenia risk in aging populations to optimize health outcomes.

We observed sex differences in the prevalence of sarcopenia, with men showing a higher prevalence compared with women. The included studies also proposed that male gender could be a likely risk factor for developing sarcopenia, which may be influenced by several biological and behavioral mechanisms. For example, hormonal differences, including lower levels of testosterone in older men and its impact on muscle mass maintenance, could contribute to higher susceptibility in aged men (28). In addition, lifestyle factors such as higher smoking behaviors in men may also play a role in exacerbating these disparities (29). Understanding these complex mechanisms is crucial for developing specific interventions to manage sarcopenia effectively in both sexes.

Regarding the factors associated with sarcopenia risk, we found that higher body fat, lower socioeconomic status, lower education, insufficient physical activity, diabetes, smoking, malnutrition, and low serum albumin levels might increase the likelihood of sarcopenia. By contrast, dietary patterns rich in anti-inflammatory nutrients, Mediterranean diet adherence, and higher mineral and vitamin intake were inversely associated with sarcopenia likelihood, which could be explained by their ability to support muscle preservation and function by providing antioxidants, reducing inflammation, and promoting muscle-building processes (30, 31). These findings underscore the multifactorial nature of sarcopenia risk. Implementing targeted interventions focused on improving nutrition, enhancing physical activity, and promoting health education may effectively mitigate the risk of sarcopenia among susceptible populations.

The overall high prevalence of sarcopenia in Iran warrants the urgent need for public health strategies focused on early detection and intervention. Our sensitivity analysis revealed that the prevalence of severe sarcopenia is quite similar to the overall prevalence, suggesting that many cases may already be in advanced stages by the time of diagnosis. However, it is important to interpret this finding with caution, as the estimate for severe sarcopenia is based on only two studies, which limits the robustness of this result. This emphasizes the need for further research and more comprehensive data to better understand the prevalence and clinical implications of severe sarcopenia in Iran. Variations in prevalence across different diagnostic criteria underline the importance of standardized diagnostic approaches to ensure consistency in identifying and managing sarcopenia. The gender disparity suggests tailored screening and intervention strategies based on gender-specific risks. Older adult individuals represent a particularly vulnerable group requiring targeted interventions to preserve muscle mass and function. Health conditions like diabetes and malnutrition implicated in sarcopenia risk highlight opportunities for integrated health management approaches. Socioeconomic factors and lifestyle choices linked to sarcopenia risk underscore the importance of holistic interventions addressing nutrition, physical activity, and socioeconomic disparities. These clinical implications emphasize the need for comprehensive, multidisciplinary strategies to mitigate the impact of sarcopenia and improve the quality of life among Iranian patients.

A limitation of this systematic review and meta-analysis was the observed high heterogeneity in our analyses. To address this, subgroup analyses revealed that factors such as sarcopenia classification and sex may partly account for this heterogeneity. Although our meta-regression analysis showed no significant impact of the different diagnostic criteria on the pooled prevalence estimates, the high heterogeneity suggests that variability in prevalence rates could be due to these different tools and highlights the need for caution when interpreting pooled data; future research should aim to standardize diagnostic criteria and reduce inconsistencies and improve comparability across studies. Overall, it is important to note that high heterogeneity is often anticipated in prevalence meta-analyses (32). Another weakness of our study was the limited data available from most Iranian provinces, and therefore, interpretation of our findings should be done cautiously; conducting additional surveys on the prevalence of sarcopenia and its associated factors across Iran could significantly enhance the current epidemiological knowledge. Moreover, we did not have access to the raw data from the included studies, preventing the possibility of conducting an individual participant data meta-analysis. Finally, due to the limited number of studies (*n* < 10), we should interpret the results of publication bias assessment and meta-regression analysis with caution.

## Conclusion

According to this systematic review and meta-analysis, the prevalence of sarcopenia is notable in Iran, especially among men and older adults. Socioeconomic factors, lifestyle behaviors, and health conditions like diabetes and malnutrition were associated with sarcopenia. Promoting physical activity, nutritional education, and addressing socioeconomic disparities are crucial steps to controlling sarcopenia risk in Iran.

## Data Availability

The original contributions presented in the study are included in the article/Supplementary material, further inquiries can be directed to the corresponding author.
