# Supplementary material for: Prevalence of, and factors associated with, sarcopenia in Iran: a systematic review and meta-analysis
Source: Front Nutr. 2025 Jan 7;11:1457768. doi: 10.3389/fnut.2024.1457768 (PMC11747721; doi:10.3389/fnut.2024.1457768)
Supplement: Supplementary file 1 [file Data_Sheet_1.PDF]

SEARCH STRATEGY

PubMed

(sarcopenia[Title/Abstract] OR sarcopenic[Title/Abstract]) AND (Iran[Title/Abstract] OR Iranian[Title/Abstract] OR Iranians[Title/Abstract])

***

Scopus

((TITLE (sarcopenia OR sarcopenic) OR ABS (sarcopenia OR sarcopenic))) AND ((TITLE (iran OR iranian OR iranians) OR ABS (iran OR iranian OR iranians)))

***

Embase

#1 sarcopenia:ti,ab OR sarcopenic:ti,ab

#2 iran:ti,ab OR iranian:ti,ab OR iranians:ti,ab

#1 AND #2 AND [embase]/lim

Supplementary Table 1. Risk of bias assessment of studies reporting the prevalence of sarcopenia in Iran, using Hoy et al.'s checklist

Study	Risk of Bias Items								
	**Target population**	**Sampling frame**	**Sample selection**	**Response rate**	**Data collection direction**	**Case definition**	**Study instrument**	**Data collection mode**	**Numerators and dominators**
Dorosty, 2016	No	No	Yes	Yes	Yes	Yes	Yes	No	Yes
Hashemi, 2016	No	No	Yes	No	Yes	Yes	Yes	Yes	Yes
Maghbooli, 2017	No	No	No	No	Yes	No	Yes	Yes	Yes
Maghbooli, 2022	No	No	No	Yes	Yes	No	Yes	Yes	Yes
Mohseni, 2017	No	No	Yes	No	Yes	Yes	Yes	Yes	Yes
Nasimi, 2019	No	No	Yes	No	Yes	Yes	Yes	Yes	Yes
Pasdar, 2022	Yes	Yes	Yes	Yes	Yes	No	Yes	Yes	Yes
Shafiee, 2020	No	No	Yes	Yes	Yes	Yes	Yes	No	Yes

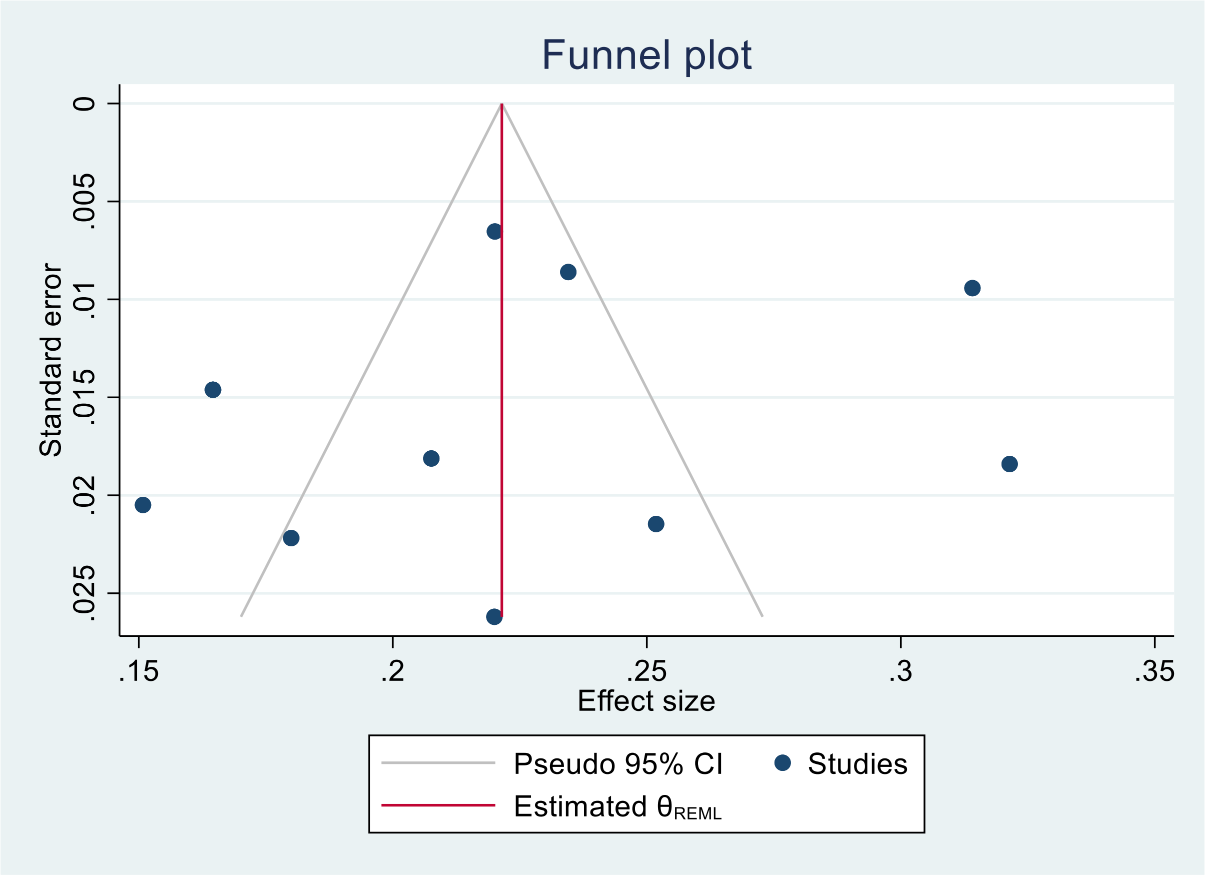

Supplementary Figure 1. Funnel plot of studies assessing the prevalence of sarcopenia in Iran

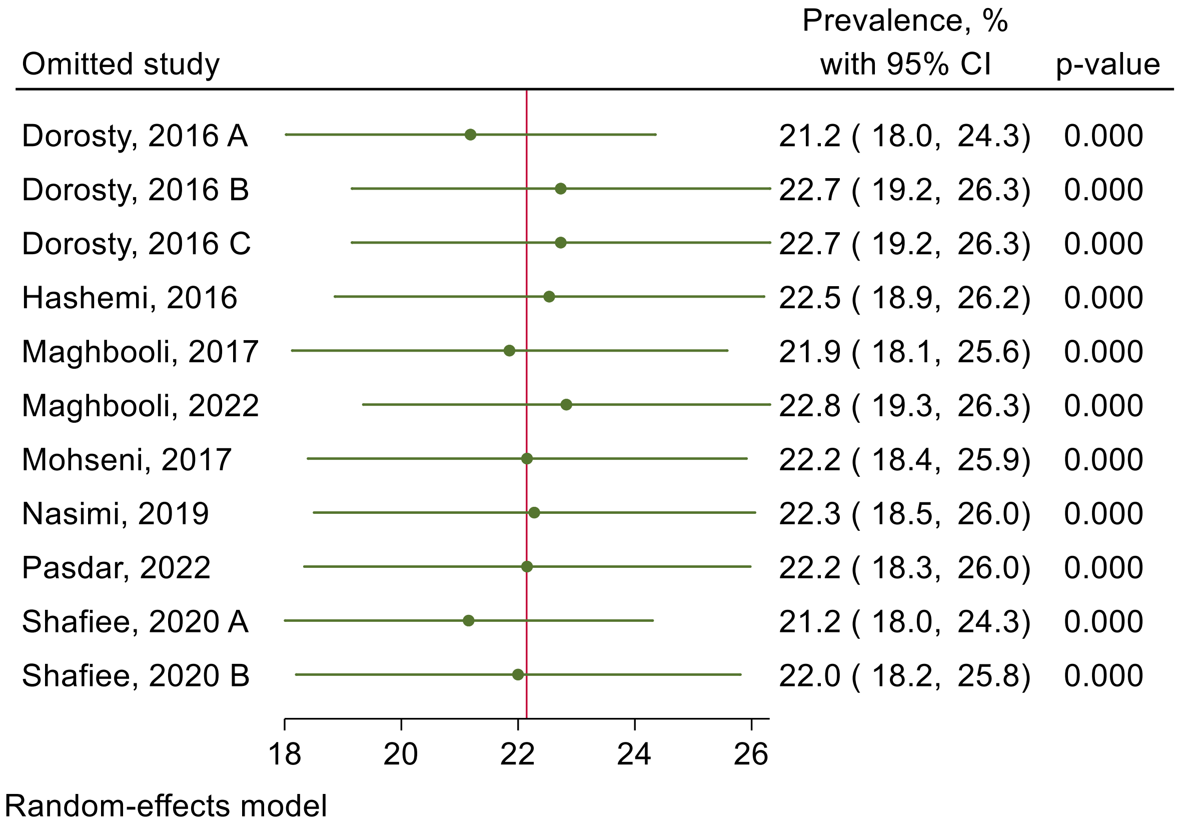

Supplementary Figure 2. Leave-one-out meta-analysis for the prevalence of sarcopenia in Iran
